# Massive hemoptysis two months after an otherwise mild SARS-CoV-2 disease (COVID-19) treated with bronchial artery embolization - A case report

**DOI:** 10.1016/j.radcr.2021.12.048

**Published:** 2022-01-15

**Authors:** Gernot Rott, Frieder Boecker, Clemens Maurer, Timur Sellmann

**Affiliations:** a Department of Radiology, Bethesda-Hospital, Heerstr. 219, Duisburg, 47053, Germany; b Institute of Clinical Radiology, Lukas-Hospital, Neuss, Germany; c Department of Pneumology, Bethesda-Hospital, Duisburg, Germany; d Department of Anaesthesiology and Intensive Care Medicine, Bethesda-Hospital, Duisburg, Germany

**Keywords:** COVID-19, Coronavirus disease 2019, Hemoptysis, Bronchial artery embolization

## Abstract

An otherwise healthy young man presented with massive hemoptysis 2 month following a mild coronavirus disease 2019 (COVID-19) and with no other identifiable cause of illness. The patient was successfully treated with bronchial artery embolization. We are strongly convinced that hemoptysis in this case was COVID-related. This unusual case of delayed COVID-related hemoptysis reveals new aspects in the understanding of mid-term and presumable auto-immune triggered effects in patients with initially only mild symptoms of the disease.

## Introduction

Within the broad spectrum of clinical manifestations of COVID-19 at least all severe cases have to be considered as a multi-systemic disease involving several organs and tissues, in particular, if not primarily the vascular system of the lung [1]. In this process, vascular involvement clinically mainly presents as pulmonary embolism. Microvascular involvement of the lung in autopsied patients, beyond thrombi, however also reveals increased pulmonary angiogenesis with foci of vascular proliferations of varying sizes and endothelial injuries [2,3]. Although hemoptysis is a relative rare presentation in COVID-19 patients, it may present even as the initial symptom of the disease [4,5,6,7].

## Case presentation

A 33-year-old man presented in our emergency ambulance with at least moderate hemoptysis persisting over three days. He had no comorbidities, took no regular medications, was a non-smoker, had not been vaccinated against COVID-19, but a history of a reverse-transcription polymerase chain reaction (RT-PCR)-confirmed SARS-CoV-2 variant B.1.1.7 infection two months ago. His only symptoms in this period had been weakness and tiredness over2 weeks, but subsequently without any clinical indication of long-COVID or post-COVID syndrome. The patient was admitted to the intensive care unit for further monitoring and therapy.

During the hospital-stay in total six RT-PCR-tests for SARS-CoV-2, four times performed with nasopharyngeal swabs and two times with bronchoalveolar lavage were carried out, all with negative result. Medical history, nearly all laboratory analysis and further investigations indicated no other apparent cause of hemoptysis. Especially inflammation parameters and d-dimers were not elevated. The only remarkable laboratory value was an elevated antinuclear antibody (ANA) titer of 1:320.

On the day of admission bronchoscopy demonstrated massive amounts of blood in the right lower lobe with diffuse bleeding. A topical vasoconstrictive agent was locally instilled.

Computed tomography angiography (CTA) of the chest only revealed small areas of subtle ground-glass opacities in the right middle and lower lobe with no other pathology (not shown).

Following a temporary clinical and bronchoscopic confirmed improvement, massive hemoptysis again occurred at night-time on day 5. Hemoglobin level meanwhile had dropped from initially 15.4 g/dl to 11.0 g/dl. The source of bleeding again was demonstrated in the right lower lobe, endobronchial balloon tamponade of the right lower bronchus was performed and bronchial artery embolization (BAE) was planned.

**Fig.1:**
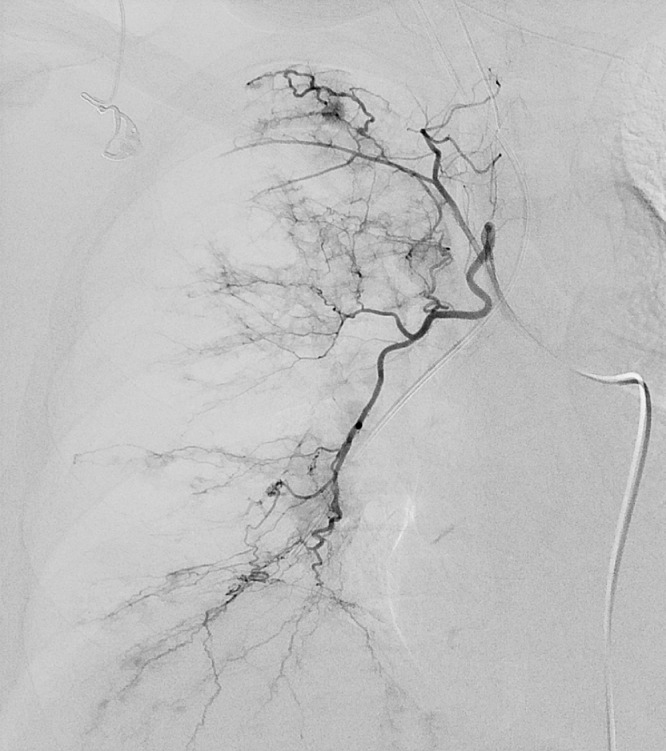
**–** Digital subtraction angiography of the mild dilated right intercostal-bronchial trunk showing disseminated tiny focal areas of patchy hypervascularization of the right lung representing inflammatory blushes.

**Fig.2:**
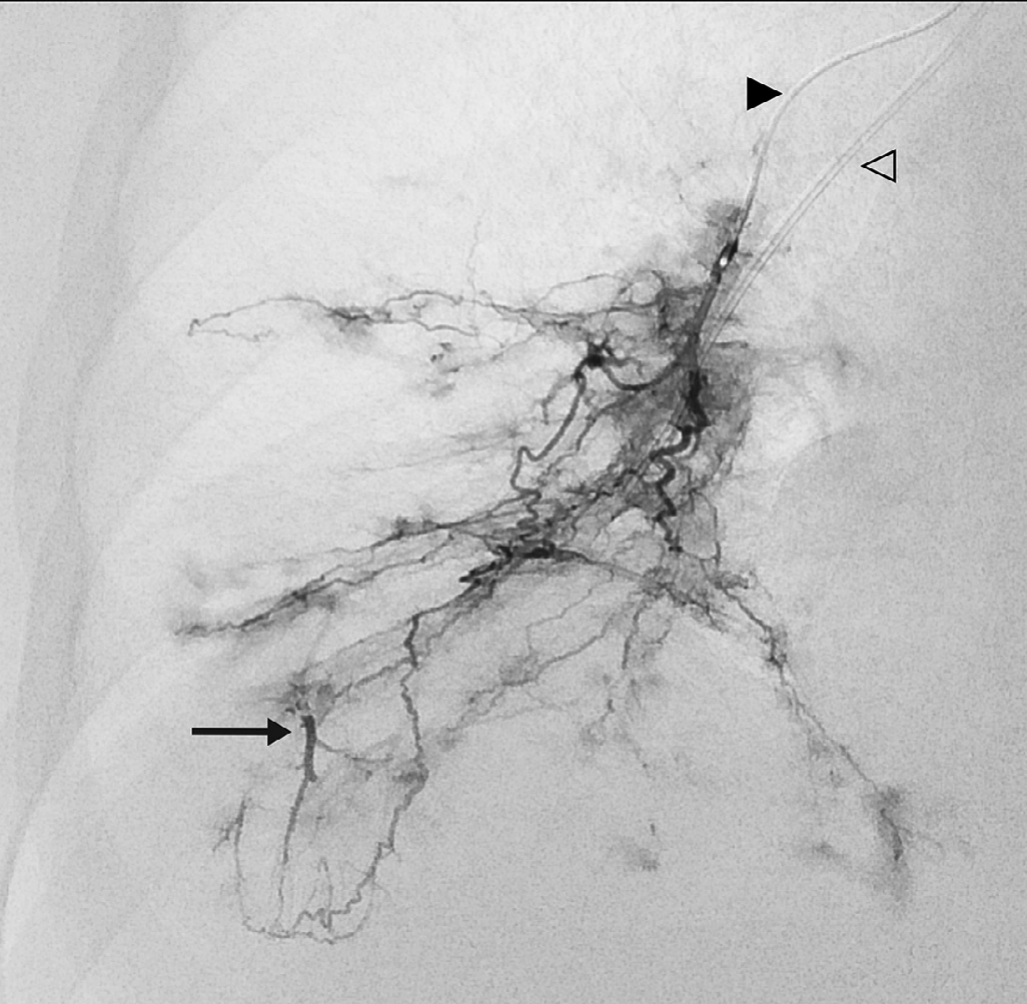
**–** Selective angiography of the right lower lobe bronchial artery with same findings as in Fig. 1 and additionally a small peripheral pulmonary artery, consistent with a bronchopulmonary shunt (arrow). Microcatheter (black arrowhead) in lower lobe bronchial artery and endobronchial balloon-catheter (white arrowhead) in lower lobe bronchus.

**Fig. 3:**
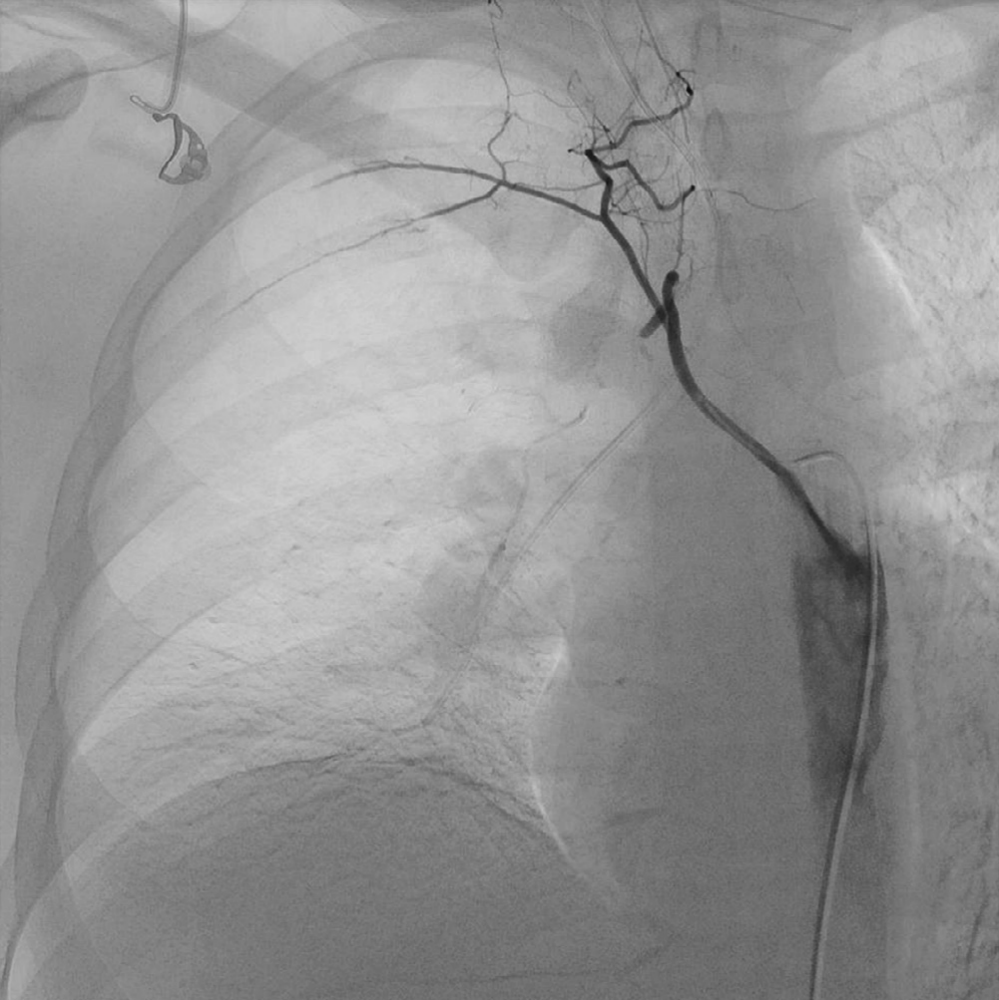
**–** Digital subtraction angiography of the right intercostal-bronchial trunk after embolization of the right bronchial artery.

The next morning, catheter angiography of the right intercostal-bronchial trunk showed slightly enlarged vessels with disseminated small focal areas of peripheral micro-hypervascularization of the whole right lung (Fig. 1). A selective angiography of the right lower lobe bronchial artery additionally identified a small peripheral bronchopulmonary shunt in the anterobasal lower lobe (Fig. 2). Embolization of the right bronchial artery was performed with use of N-butyl-2-cyanoacrylate (NBCA) (Fig.3).

The postinterventional course was uneventful, no further hemoptysis occurred, hemoglobin level rose and the patient was discharged five days after the embolization with no complaints.

## Discussion

Massive hemoptysis has been described as a rare symptom and even as the first presentation of COVID-19 in case reports or small case series, in any concrete event, however, all in patients with either severe disease or relevant comorbidities, such as chronic obstructive lung disease or in active smokers [4,5,6,7]. This observation is contrasted by the study of Lapostolle et al. [8] of a large cohort of COVID-patients with only mild-to-moderate symptoms in the Paris area treated with outpatient management, which revealed a hemoptysis-rate of 3%. Consequently we may assume that hemoptysis is not a very rare symptom in COVID-19, even in patients with only mild symptoms.

Lung changes of autopsied COVID-patients sufficiently explain why hemoptysis in patients with severe clinical course of the disease might occur [2,3]. The fundamental findings of Ackermann et al. on lung microangiopathy of COVID-patient not only showed microthrombi with involvement of all segments of the vasculature but also severe endothelial injury with disrupted endothelial cell membranes and significant new vessel growth with intussusceptive angiogenesis [3].

Our patient had the medical history of a mild symptomatic COVID-19 2 months ago and, in addition, the only remarkable laboratory parameter of an elevated ANA-titer. Elevated ANA-titers are frequently seen in COVID-patients and thought to be associated with the severity of illness and the extent and chronicity of autoimmune symptoms [9]. A high frequency of clinically relevant autoantibodies has been observed in acute and convalescent samples from COVID-patients, both in patients from intensive care units as well as in patients with only mild infections. Their frequency suggests that autoantibodies may contribute to the long-term consequences of COVID-19 [9,10,11]. ANA-positive patients seem to have a worse prognosis with respect to COVID-19 [10].

In addition, catheter angiography of our patient revealed relative subtle but disseminated changes of the whole right lung, indicating not a focal but a disease of the whole organ, as expectable in a systemic disease or may be seen in pneumonia.

Since history, clinic, laboratory tests and imaging of the patient all showed findings as regularly observed in COVID-19, the case presented definitely cannot be assessed as cryptogenic hemoptysis [12].

Hemoptysis in COVID-patients with much more extended lung changes or severe acute respiratory syndrome has been treated with BAE in a few cases [13,14]. The case of a patient “suffering from long-COVID” with hemoptysis eight months after the diagnosis and BAE has recently been published, however with a pseudoaneurysm of a bronchial artery as the morphological substrate of bleeding [15]. To the best of our knowledge, there is no published case of delayed massive hemoptysis following months after an only mild symptomatic COVID-19 or without a pseudoaneurysm.

We are undoubtedly convinced that hemoptysis in our patient had the same cause as detected in patients with severe COVID-19, namely proliferative changes with endothelial damages and microthrombi in small vessels probably triggered by autoimmune processes, in this case, however, as a delayed impact of COVID-19. This report adds a new clinical aspect in the understanding of post-acute to mid-term effects of COVID-19 on microvascular changes of the lung in patients even with only mild symptoms.

## Patient consent

The authors declare that this report does not contain any personal information that could lead to the identification of the patient. Formal consent of the patient was not required.

## Author contributions

All authors attest that they meet the current International Committee of Medical Journal Editors (ICMJE) criteria for Authorship.

## Ethics human rights

The authors declare that the work described has been carried out following the Declaration of Helsinki of the World Medical Association revised in 2013 for experiments involving humans.

## Declaration of Competing Interest

The authors have no conflicts of interest
